# Quantification of tongue mobility impairment using optical tracking in patients after receiving primary surgery or chemoradiation

**DOI:** 10.1371/journal.pone.0221593

**Published:** 2019-08-27

**Authors:** K. D. R. Kappert, M. J. A. van Alphen, L. E. Smeele, A. J. M. Balm, F. van der Heijden

**Affiliations:** 1 Head & Neck Oncology and Surgery, Netherlands Cancer Institute, Amsterdam, The Netherlands; 2 Robotics and Mechatronics, University of Twente, Enschede, The Netherlands; 3 Oral and Maxillofacial Surgery, Amsterdam UMC, University of Amsterdam, Amsterdam, The Netherlands; University of Wisconsin, UNITED STATES

## Abstract

**Purpose:**

Tongue mobility has shown to be a clinically interesting parameter on functional results after tongue cancer treatment which can be objectified by measuring the Range Of Motion (ROM). Reliable measurements of ROM would enable us to quantify the severity of functional impairments and use these for shared decision making in treatment choices, rehabilitation of speech and swallowing disturbances after treatment.

**Method:**

Nineteen healthy participants, eighteen post-chemotherapy patients and seventeen post-surgery patients were asked to perform standardized tongue maneuvers in front of a 3D camera system, which were subsequently tracked and corrected for head and jaw motion. Indicators, such as the left-right tongue range and the deflection angle with the horizontal axis were extracted from the tongue trajectory to serve as a quantitative measure for the impaired tongue mobility.

**Results:**

The range and deflection angle showed an excellent intra- and interrater reliability (ICC 0.9) The repeatability experiment showed an average standard deviation of 2.5 mm to 3.5 mm for every movement, except the upward movement. The post-surgery patient group showed a smaller tongue range and higher deflection angle overall than the healthy participants. Post-chemoradiation patients showed less difference in tongue ROM compared with healthy participants. Only a few patients showed asymmetrical movement after treatment, which could not always be explained by T-stage or the side of treatment alone.

**Conclusion:**

We introduced a reliable and reproducible method for measuring the ROM and to quantify for motion impairments, that was able to show differences in tongue ROM between healthy subjects and patients after chemoradiation or surgery. Future research should focus on measuring patients with oral cancer pre- and post-treatment in combination with the collection of detailed information about the individual tongue anatomy, so that the full ROM trajectory can be used to identify changes over time and to quantify functional impairment.

## Introduction

Head and neck cancer is the sixth most frequently occurring cancer worldwide. Carcinoma of the tongue and base of the tongue account for about 20% of all head and neck cancers[1], and incidences are rising, particularly in the oropharynx due to HPV infections [2]. Surgery is the most preferred treatment for tongue carcinomas, whereas base of tongue carcinomas are mostly treated by organ sparing radiation with or without concurrent chemotherapy[3]. In advanced cases, both treatments might seriously affect the mobility of the tongue, resulting in impaired speech, swallowing, or mastication[4,5].

The current understanding of post-treatment tongue function in clinical practice is based on anatomical and physiological reasoning and personal experience. Surgical treatment of lateral tongue carcinoma often leads to asymmetrical tongue movements[6,7]. With increasing T stage this impairment becomes more outspoken and is accompanied by deterioration of speech quality and mastication function[8–10]. The organ sparing surgical chemoradiation is usually the preferred treatment for base of the tongue tumors, but in advanced cases this modality may also lead to serious functional deficits with more impact on swallowing than surgery of the mobile tongue[3,11]. Currently, it is not possible to accurately predict the functional impairments at an individual level, therefore clinical decision making, which implements expected functional sequelae, remains mainly dependent on the personal experience of the treating physician.

Tongue mobility has shown to be a clinically interesting parameter on functional results and can be objectified by measuring the Range Of Motion (ROM) [12–15]. Reliable measurements of impaired ROM would enable us to quantify the severity of functional impairments and use these for shared decision making in treatment choices, rehabilitation of speech and swallowing disturbances after treatment[15,16]. In addition, the ROM and other characteristics of the 3D trajectory of the tongue can be used as an input feature for biomechanical models aimed at predicting consequences of treatment [17–19]. Classical imaging techniques such as video fluoroscopy and ultrasound can visualize the tongue in a sagittal slice to evaluate the shape of the tongue in a 2D plane, but are not aimed at tracking the 3D position of the tongue [20–22]. Although MRI techniques are rapidly advancing, they are still not able to capture the 3D motion of the tongue[23–26]. Electromagnetic articulography (EMA) is a reliable technique to measure the 3D shape of the tongue over time and has, over the past decades, been used in research focussed at speech swallowing and mastication function[27–32]. It is, however still a very expensive and complicated procedure that is not comfortable for the patients.

In 2016, our research group published a paper about a triple camera set up to assess 3D ROM information of the tongue tip as a fast, secure and accessible alternative for classical imaging techniques and EMA [15]. We showed that this was a reliable tool (intraclass correlation of over 0.9) for impaired tongue mobility after a partial glossectomy. By manually selecting four landmarks on the head and one on the tip of the tongue for every camera position, the distance between the interdental papilla and the tip of the tongue was determined. Using this system we were able to show impaired mobility to the contralateral side of the resection in glossectomy patients[15].

Although the triple camera set-up was sufficient for pointing out differences between patients, the technique still had some limitations: a) The maximum 3D deflection of the tongue during a specific maneuver was calculated by manual selection of the tongue tip in two 2D videos, thereby the full 3D trajectory information of the tongue tip was not used. b) For a left-right maneuver, the Euclidian distance was used as a measure for the ROM. When the participant showed a deviation, other than horizontal, this method tended to overestimate the horizontal deflection. c) Jaw movements were not accounted for, and d) Due to self-occlusion, the tip of the tongue was often not visible. In these cases, the ROM would be inaccurate or even incorrect.

In this study, we describe a 3D tracking tool for measuring the complete trajectory of tongue tip movements to address the aforementioned limitations of the triple camera set-up. From this trajectory, we can derive indicators which are potential quantitative measures for the mobility impairment of the tongue after treatment. This comprehensive approach would be more suitable for clinical use and input for biomechanical tongue models. The research questions of this study are:

1. What are possible indicators using a 3D tracking tool for impaired tongue mobility after tongue cancer treatment?
2. Are these indicators reliable and reproducible?
3. Can the asymmetry of the tongue mobility be quantified?

## Materials and methods

### Participants

To determine if the improved method can be used to objectively determine asymmetry, we included a total of 57 participants between June 2017 and December 2018. Nineteen healthy participants, Nineteen tongue carcinoma patients (tumor stages T1-T3) who had undergone a partial glossectomy followed by primary closure of the defect, and Nineteen patients with a carcinoma of the tongue base (tumor stages T1 -T4) who had been treated solely with chemoradiation. All healthy participants were at the age of 18 or older and did not have any history of oral cancer or other diseases that might influence the mobility of the tongue. In the two patient groups, ROM was measured at least six months after treatment. The two patient groups will later be referred to as the post-surgery and the post-chemoradiation group, respectively.

All procedures performed in studies involving human participants were in accordance with the ethical standards of the medical ethical committee of the Netherlands Cancer Institute and with the 1964 Helsinki declaration and its later amendments or comparable ethical standards. A written Informed consent was obtained from all individual participants included in the study and was approved by the medical ethical committee of the Netherlands Cancer Institute (ref: N17SWU).

### Experimental setup

**Fig 1:**
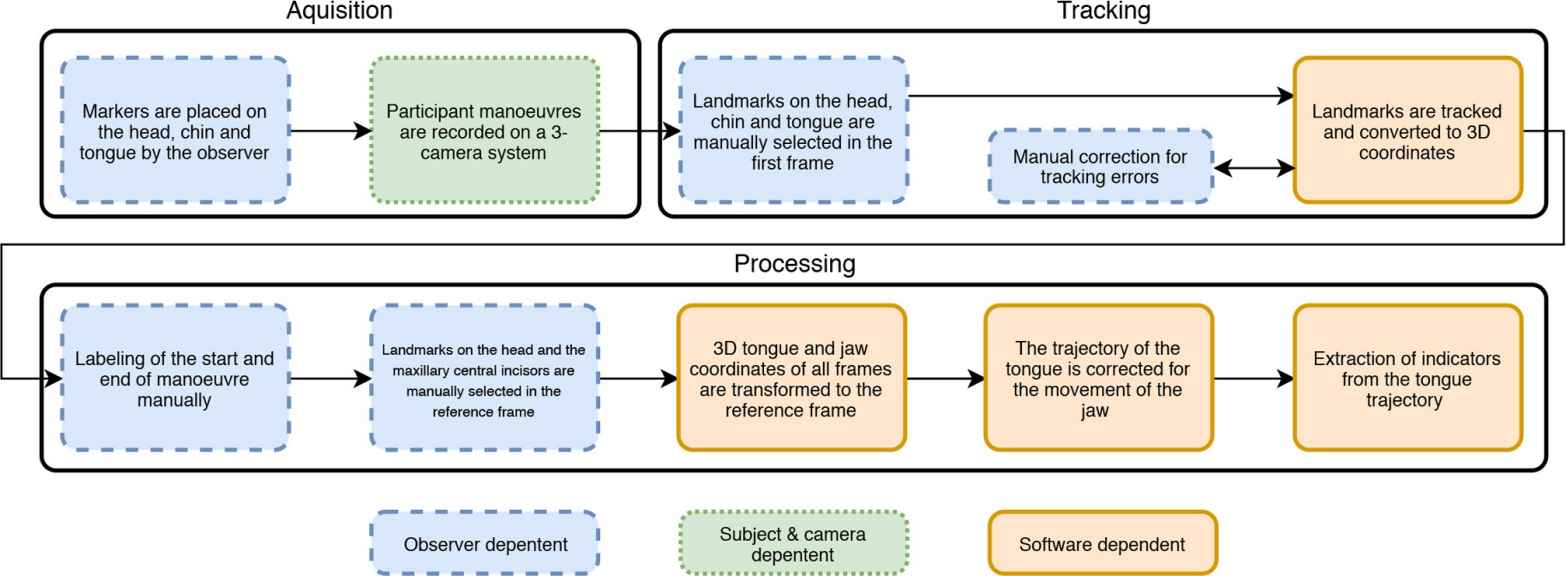
Flowchart, summarizing the measurement steps and data processing. The color and outline refer to the actuator responsible for potential variation during a specific part of the process.

The experimental workflow includes acquisition, tracking, processing, and extraction of indicators for the tongue mobility impairment, which are explained in the next paragraphs and are summarized in Fig 1.

#### Acquisition

**Fig 2:**
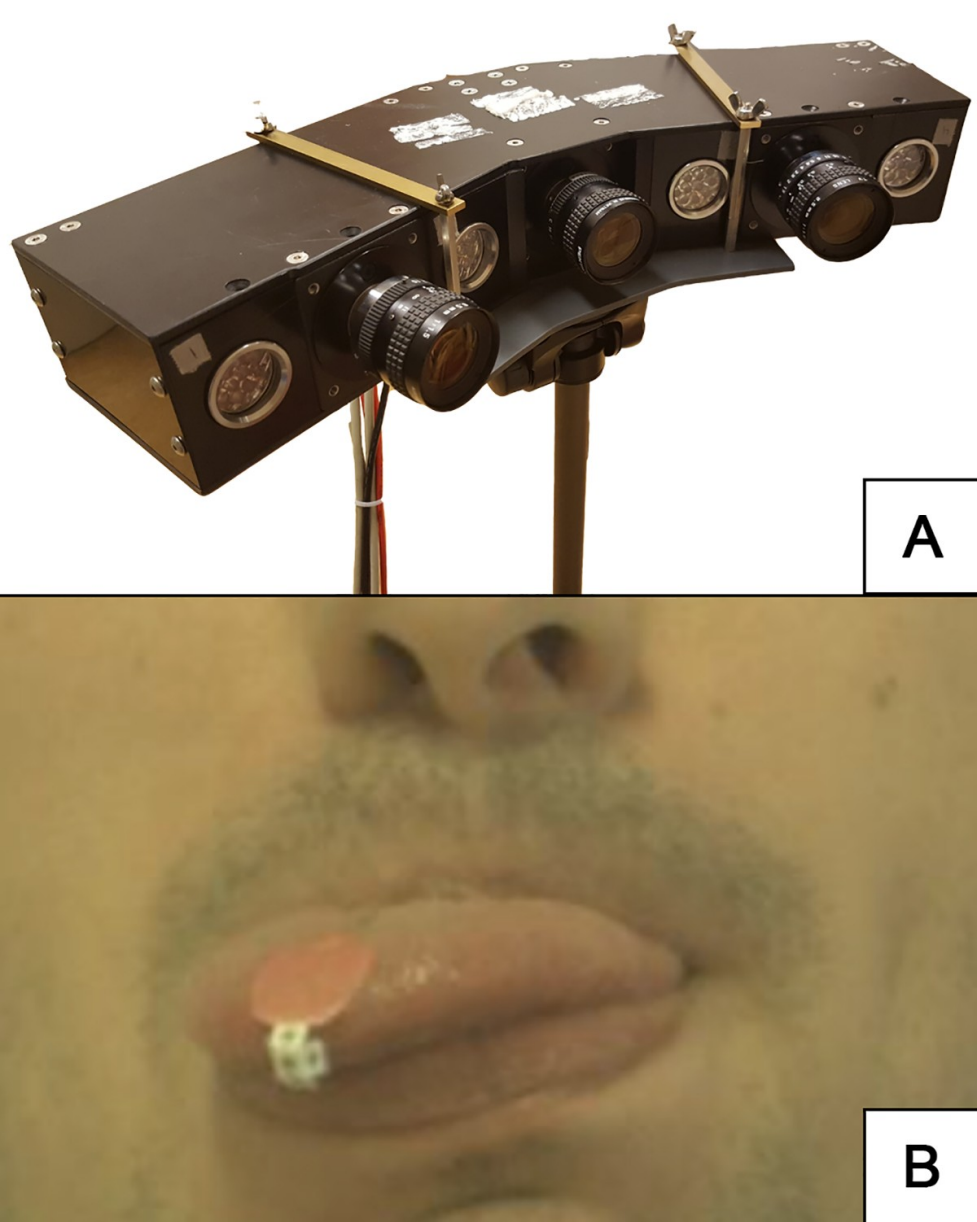
Requisites for ROM measurement. (a) The triple camera system. (b) The tongue marker, 3D paper cube, placed on the tongue tip of a healthy participant.

**Fig 3:**
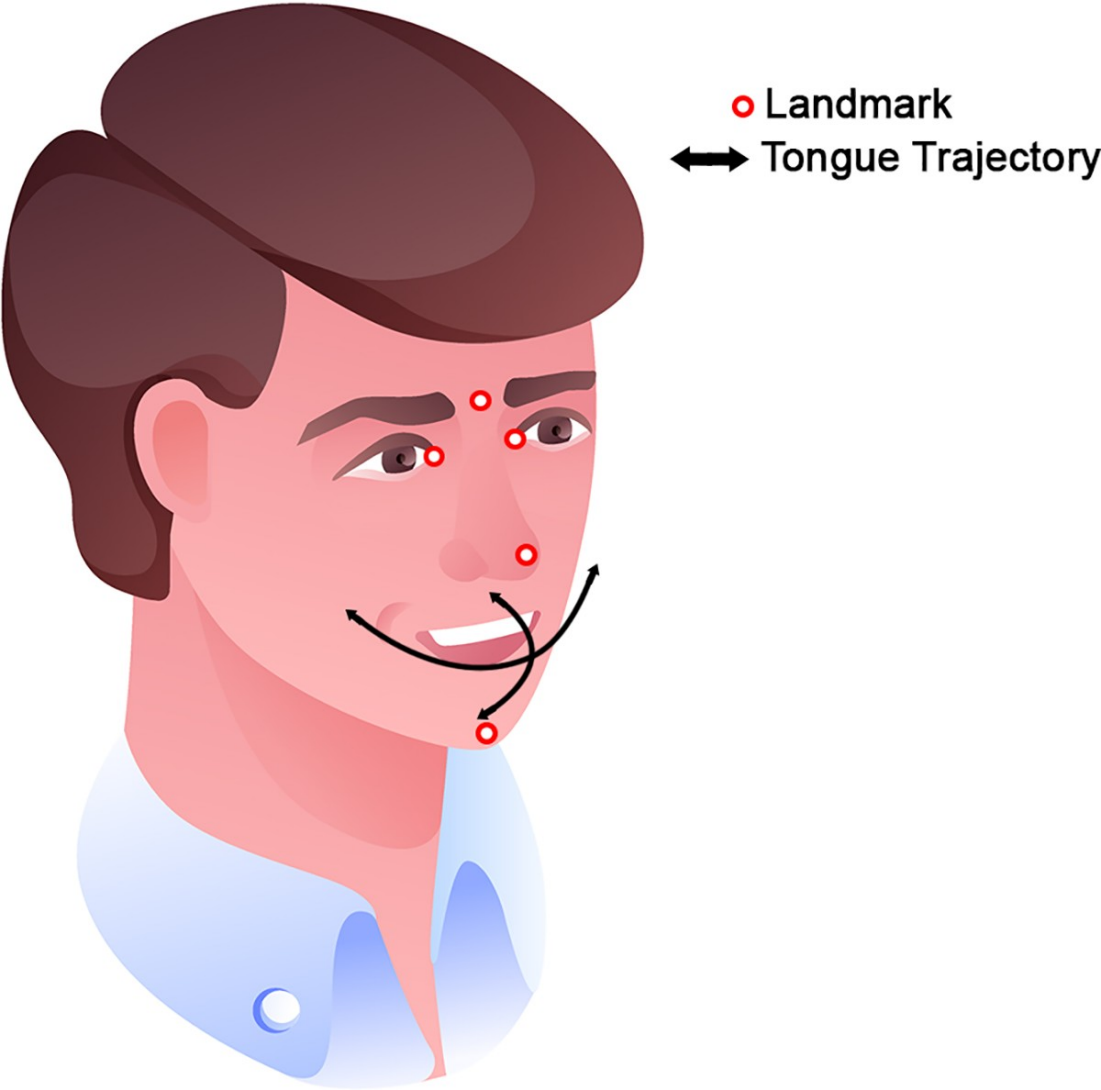
An illustration of a participant’s head and all landmarks and tongue maneuvers. The tongue maneuvers are visualized as black arrows. Markers on were placed the head, chin and nose of the participant in order to track them. The caruncles of the eyes do not require external markers as they are a distinguishable facial feature. (Illustration designed by Vectorpouch / Freepik).

To measure the position of the tongue tip, a custom-made 3D camera setup was used. The setup consists of three Basler^®^ av1000-100gc 100fps cameras, horizontally aligned, and targeted at the tongue with an angle of 20⁰ from each other (Fig 2A). The cameras were calibrated using 300 video frames of a checkerboard image and the Matlab^®^ stereo-calibration tool. To enable tracking of the tongue tip, a paper marker was designed (Fig 2B). This marker-design features a 3D paper cube to ensure that the marker is visible from every angle. Additional markers were placed on the glabella, apex of the nose and mental region to enable tracking of the head and jaw (Fig 3). The caruncles of the eyes did not require external markers as they are distinguishable landmarks.

After the paper marker was placed on the tongue tip by the observer, the recording was started and participants were asked to perform three different maneuvers:

- Protrusion left to right with the tongue
- Protrusion down to up with the tongue
- Showing the maxillary central incisors

The participants were instructed to always protrude the tongue as far as possible in all directions (Fig 3).

#### Tracking

A user interface was developed to extract and process the ROM trajectory from the three videos cameras. First, the locations of six landmarks were selected: the caruncles of the eyes, glabella, nose point, tongue point and mental region (Fig 3). Using the Lucas Kanade tracking algorithm implemented by Matlab (MatWorks, 2018b) the six points were tracked until the end of the video. Manual interference was possible to adjust for tracking failures. Using the camera calibration parameters, calculated by the stereo-calibration tool in Matlab (MathWorks, 2018b), the 3D positions of all tracked makers over time were reconstructed. The trajectory of the tongue tip was smoothed and equidistantly resampled. Time stamps were added manually to label the start and end of the maneuvers.

#### Processing

The 3D trajectory of the tongue tip was processed in order to compensate for head movements. For this purpose, a reference video frame wherein the maxillary central incisors were visible was chosen. In this reference frame five points were selected: the caruncles of both eyes, in between the crowns of the maxillary central incisors, the marker on the glabella and the marker on the apex of the nose. For each video frame *i*, these points were used in a Procrustes algorithm to obtain the 4x4 transformation matrix ^*ref*^**T**(*i*) that represents the 3D pose of the head in video frame *i* relative to the 3D pose of the head in the reference video frame. The transformation matrices ^*ref*^**T**(*i*) were applied to the reconstructed 3D positions of the tongue tip and jaw so that they are all expressed in the single coordinate system, which is associated with the reference video frame.

**Fig 4:**
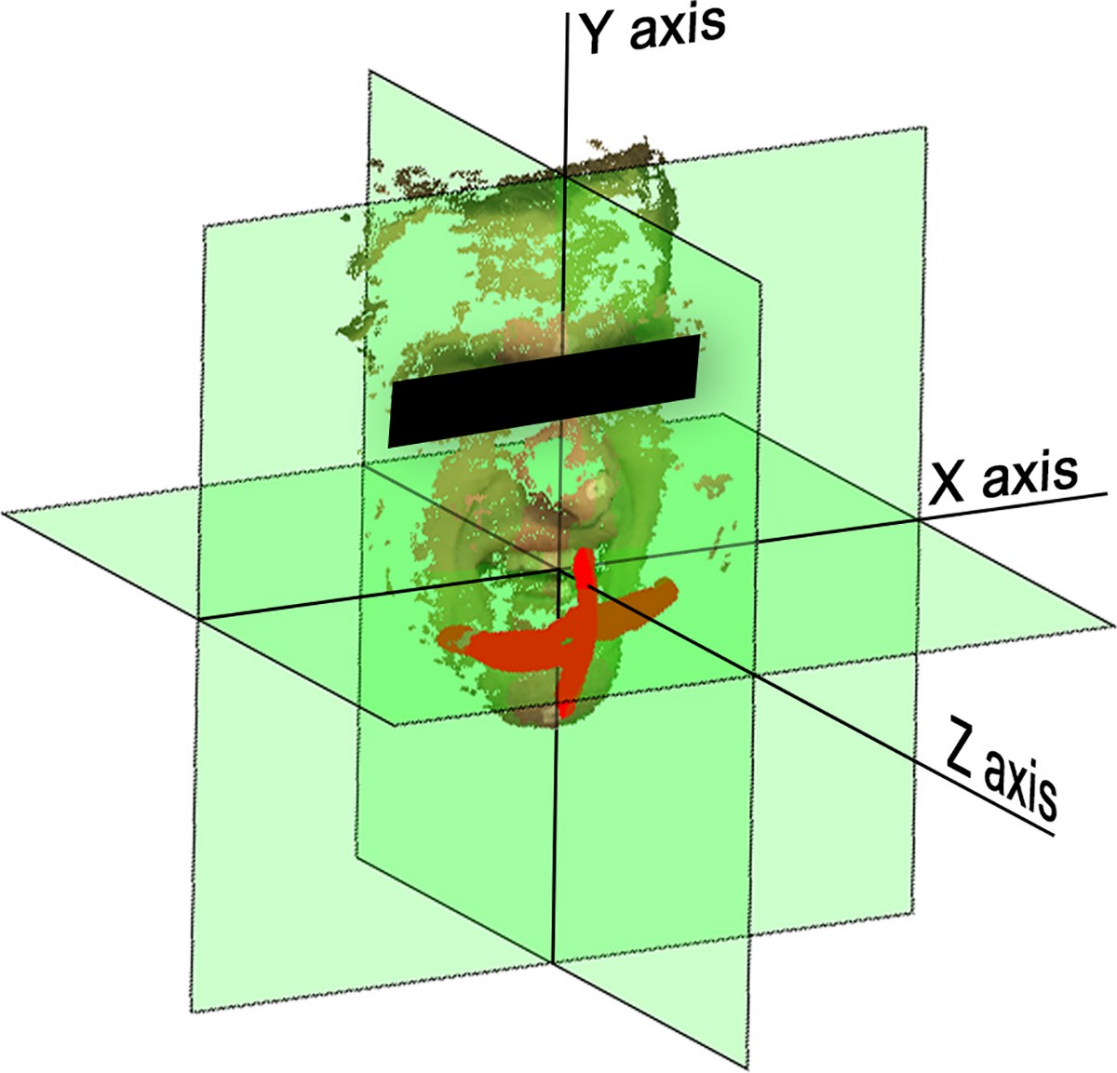
A 3D reconstruction of a participant and its tongue trajectory. The tongue trajectory represented in red. The planes of the coordinate system are transparent green. A point cloud of the face is plotted as a reference for the location of the head.

The origin of this reference coordinate system was set at the junction between the crowns of the maxillary central incisors in the reference video frame (Fig 4). The X-axis was aligned with the caruncles and the Y-axis was therefore positioned in-between the caruncles and perpendicular to the X-axis. The Z-axis was placed perpendicular to the plane formed by the caruncles and the junction between the maxillary central incisors (Fig 4).

Additionally, the trajectory of the tongue tip was corrected for jaw movement. During the down-up movement, the video frame wherein the tongue marker was closest to the horizontal plane (X-0-Z plane) was chosen as the reference video frame. The position of the jaw marker in this frame was used as a reference for jaw movement. The relative displacement of the jaw, seen from this reference point, was subtracted from the tongue tip trajectory.

#### Indicators for tongue mobility impairment

**Fig 5:**
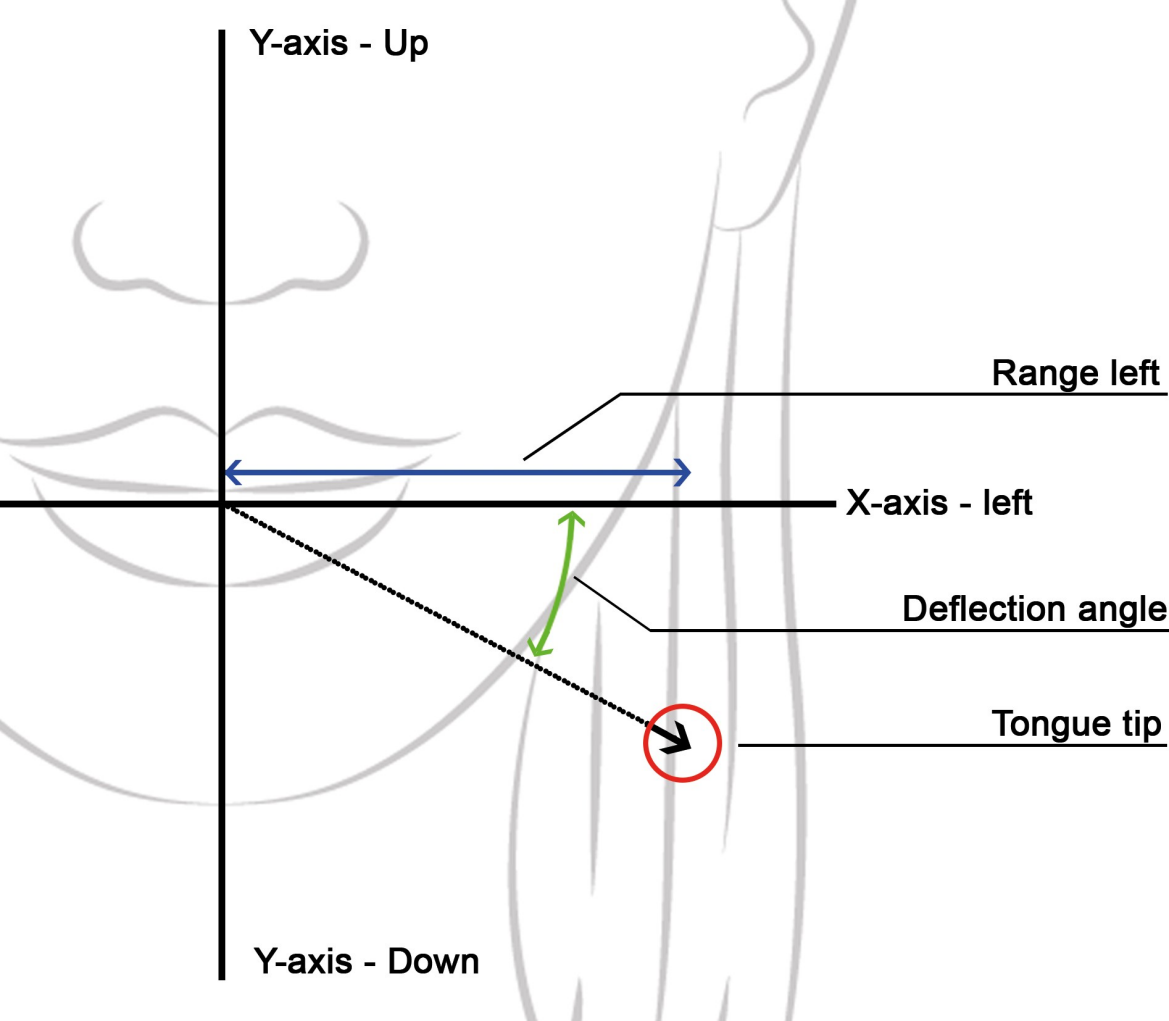
**Definition of the tongue’s range (blue) and deflection angle (green) to the left side.** The definition is based on the projection of the tongue tip (red circle) on the XY plane. The right side is a mirrored version of the left side. (background designed by Freepik).

To interpret the resulting 3D tongue tip trajectory, aspects of this trajectory need to be translated into indicators. The following indicators were extracted from the tongue tip trajectory:

- Range *R*_*l*_ and *R*_*R*_: the maximal deviation of the tongue tip in the positive and negative X direction, while performing the left to right protrusion respectively (Fig 5). This is, effectively, the extreme position of the tongue tip projected on the horizontal plane. The range of the up and down movement, *R*_*u*_ and *R*_*d*_, is defined likewise in the Y direction. The range *R*_*f*_ of the forward protrusion is defined in the Z direction.
- Deflection angles to the right and the left (*φ*_*r*_ and *φ*_*l*_): Patients who underwent surgery often have difficulties to move the tongue tip to the desired position. A way to express this is by calculating the angle between the axis of the instructed direction (X-axis for left and right) and the line from the tongue tip at maximum range to the origin over the XY plane (Fig 5).

### Validation of the method

The performance of this method depends on the variation that is induced by both the participant, the observer, the camera system and the software (Fig 1). The camera system was already validated in a previous study [15]. The root mean square error, for selecting a marker in 3D space, was 0.73 mm, which was estimated using a leave-one-out method.

#### Intrarater and interrater reliability

The observer plays a key role in some important parts of the process. It is essential that landmarks are selected and tracked in a reproducible way by the observers. Using the intra- and interrater reliability of two observers, expressed in the Intraclass Correlation Coefficient (ICC 2.1 & 3.1), we determined whether the manual interference in the tracking and processing steps is reliable. The ICC of the range and deflection angle indicators are measured using nine healthy participants. An ICC of more than 0.75 is considered an excellent agreement, and an ICC less than 0.4 a poor agreement. This was computed using SPSS (Version 25, IBM, 2018).

#### Repeatability of the indicators with and without jaw compensation

The main outcomes of our method are the range and deflection angle indicators. The repeatability of the indicators was assessed by measuring and processing a single healthy participant five times under the same conditions. The mean (μ) and the standard deviation (σ) are calculated with and without jaw-movement compensation for every maneuver. To compare standard deviations between maneuvers the coefficient of variation (c_v_) is calculated for every indicator using:

$$c_{v=}\frac{\mu }{\sigma }$$ (1)

#### Comparison with clinical expectations

Since we only have healthy or post-treatment measurements, we can only focus on the asymmetry of a participant and the differences between groups. Therefore, the total range and deflection angle are calculated:

The total range from left to right:

$$\begin{array}{c} R_{total}=R_{r}+R_{l} \end{array}$$ (2)

The combined deflection angles from left and right:

$$\varphi_{total}=\varphi_{r}+\varphi_{l}$$ (3)

To emphasize the asymmetry, the difference between the ipsilateral and contralateral deflection angle (*φ*_*ips*_ and *φ*_*contra*_, respectively) is calculated:

$$\begin{array}{c} \varphi_{dif}=\varphi_{ips}-\varphi_{contra} \end{array}$$ (4)

Where *φ*_*ips*_ equals either *φ*_*r*_ or *φ*_*l*_ depending on which side was affected.

The tumor stage (T-stage) is widely used as a parameter to categorize tumors and to differentiate patients regarding functional loss[33]. Therefore, asymmetry of the tongue range to the left and right will be compared between the two patient groups and tumor stage. Before the ranges can be compared, they have to be normalized:

$$R_{ips,normed}=\frac{R_{ips}}{R_{total}}R_{contra,normed}=\frac{R_{contra}}{R_{total}}$$ (5)

Where *R*_*ips*_ equals either *R*_*r*_ or *R*_*l*_ depending on which side is affected. The ratio between normalized tongue ranges can be calculated by:

$$\begin{array}{c} R_{dif}=\frac{R_{ips}-R_{contra}}{R_{total}} \end{array}$$ (6)

## Results

### Intrarater and interrater reliability of tracking and processing

The mean intra- and interrater reliability for the range and deflection angle are well above the ICC of 0.9 for all the measurements (see Table 1).

**Table 1:** Intra- and interrater correlation coefficients for the range and deflection angle of nine healthy participants.

	Range	Deflection angle
**Intrarater (Jaw-comp) ICC: 3,1**	0.97 (0.95–0.99)	0.95 (0.90–0.97)
**Interrater (Jaw-comp) ICC: 2,1**	0.96 (0.93–0.98)	0.92 (0.84–0.96)

### Repeatability of the indicators with and without jaw compensation

**Fig 6:**
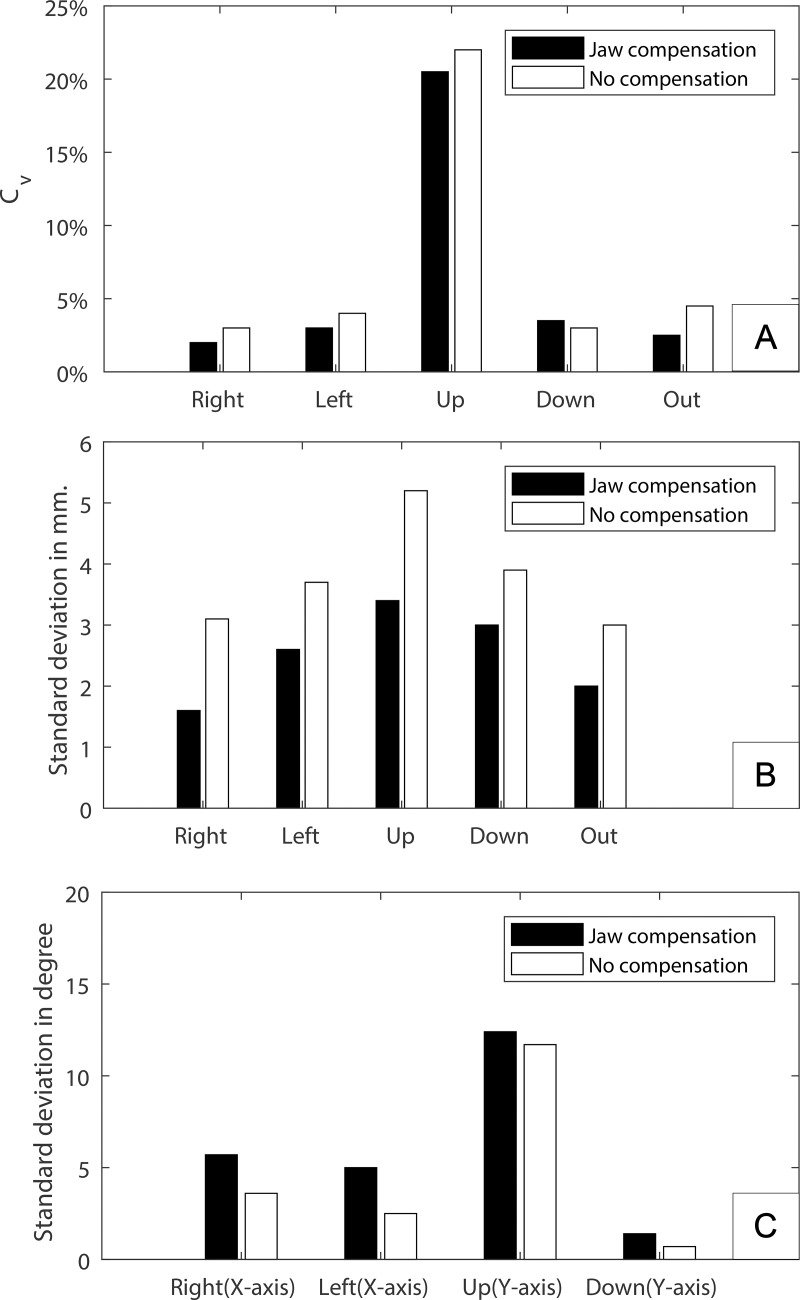
Repeatability of the tongue’s range and deflection angle indicators. Indicators with (black) and without jaw-movement compensation (white) are shown in bar plots in their specific unit measure. (a) the coefficient of variation (C_v_) of the tongue range in mm. (b) standard deviation (σ) of the tongue range in mm. (c) standard deviation (σ) of the tongue’s deflection angle in angular degrees.

The mean coefficient of variation (c_v_) of the range and the σ for both the range and deflection angle are shown in Fig 6. The measured ranges, excluding the up-maneuver, show a c_v_ of about 7% (σ of 3.5 mm) which, by adding the jaw-movement compensation, decreases to about 5,5% (σ of 2.5 mm). The up movement could not be reproduced as well as the other maneuvers; even though the σ of the up maneuver is only about one mm larger than the other maneuvers, the range is very small, which results in a relatively large error (c_v_ of 40%). The deflection angle also follows a similar pattern with the exception that the s is slightly larger when using jaw-movement compensation.

### Comparison with clinical expectations

#### Participant characteristics

Two post-surgery patients were excluded. One patient could not understand the instructions and the other had undergone a re-resection that was not previously known by the researcher. One chemoradiation patient was excluded because the marker on the mental region was not visible in many of the video frames. No healthy participants were excluded.

Eventually, we included 19 healthy, 17 post-surgery and 18 post-chemoradiation participants. The characteristics of these groups are summarized in Table 2.

**Table 2:** Participant characteristics.

Group	Median age (Range)	Tumor location L/R	Tumor stage T 1/2/3/4	Time after treatment in weeks
**Healthy participants**	53 (23–71)	-	-	-
**Partial surgery (mobile tongue)**	65 (51–84)	6/11	7/9/1/0	162(43–310)
**Post-chemoradiation (base of the tongue)**	65 (48–79)	11/7	4/7/3/4	182(21–706)

#### Comparison with clinical expectations

**Fig 7:**
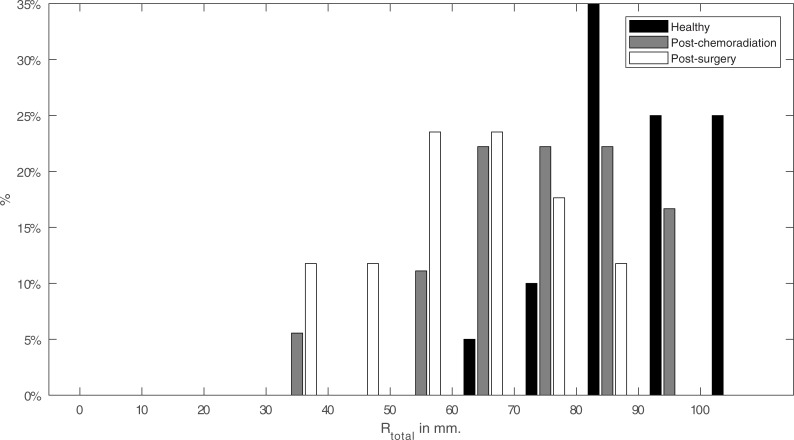
The total range of the tongue (*R*_*total*_) for healthy, post-chemoradiation and post-surgery participants. The graph shows the percentage of participants that exhibit a certain range of tongue motion when moving from left to right within a specified interval.

In Fig 7 the total range from left to right (*R*_*total*_) of all participants are expressed in bar plots. In the top Fig, the range is divided into bins of 10 mm for every participant group. The normalized counts per bin are expressed in percentages. The healthy participants clearly have the largest total range with the highest percentage around 85 mm whereas the post-surgery patients peak around 60 mm. Post-chemoradiation is right in between those two groups.

**Fig 8:**
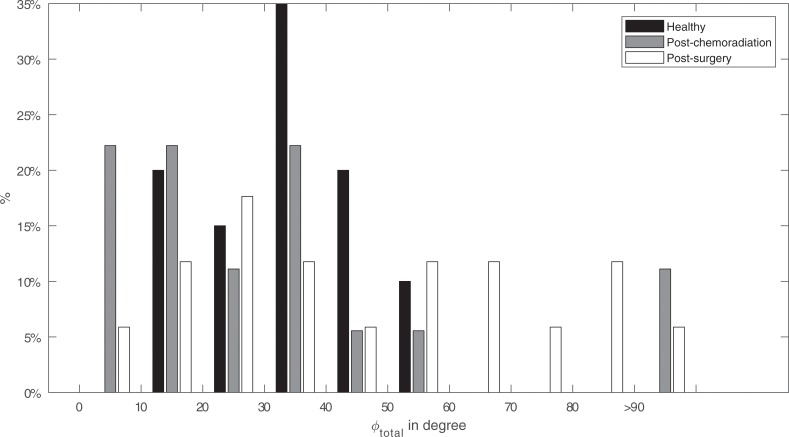
The total deflection angle of the tongue (*φ*_*total*_) for healthy, post chemoradiation and surgery participants. The graph shows the percentage of participants that exhibit a total deflection angle for the left and right movement within a specified interval.

We observe an inverse relationship if we compare the size of the total deflection angles (*φ*_*total*_) of the left and the right maneuver (Fig 8): the patients have more difficulties moving the tongue tip horizontally to the left or to the right than the healthy participants.

**Fig 9:**
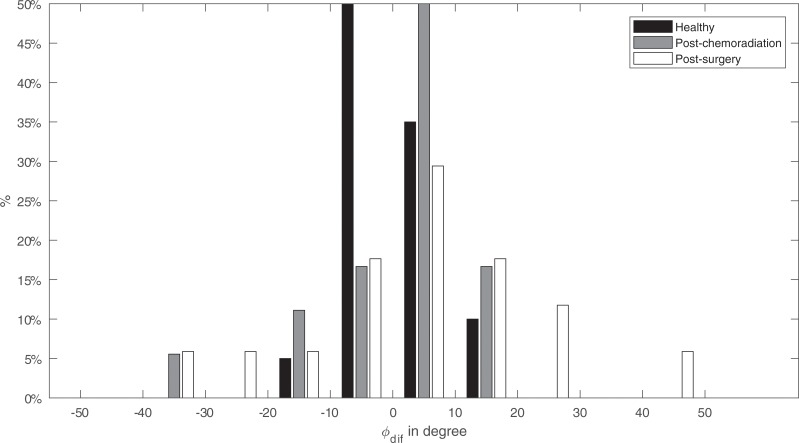
The difference between Ipsilateral deflection angle and contralateral deflection angle (*φ*_*dif*_). *φ*_*dif*_ is divided in bins of 10 angular degrees. For healthy participants, this is the difference between left and right deflection angle.

Without preoperative measurements, the asymmetry between the ipsilateral and contralateral movements, *φ*_*dif*_ and *R*_*dif*_, is a more suitable measure for impairment than the total distance or angle. For comparison, the difference between left and right among healthy participants are shown in the upcoming Figures. Fig 9 shows that most of the healthy participants express fairly symmetric behavior in moving tongue to the left and right at the same deflection angle. While most patients show this same symmetric behavior, about one-third of both patient groups show an asymmetry between the ipsilateral and contralateral angle (*φ*_*dif*_) of more than 10 degrees.

**Fig 10:**
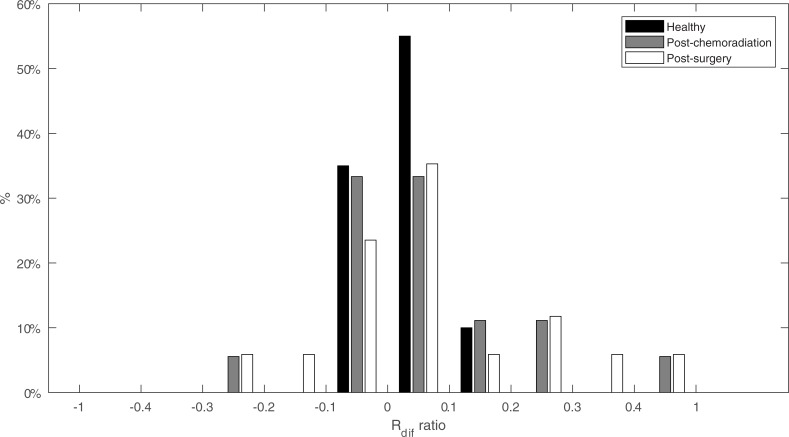
Normalized difference between ipsilateral and contralateral range (*R*_*dif*_). *R*_*dif*_ is divided in bins of 0.1 on a -1 to 1 scale. For healthy participants, this is the difference between left and right deflection range.

When looking at the difference between the normalized ipsilateral and contralateral range (*R*_*dif*_), we see a pattern that is comparable to the deflection angle (Fig 10). While most of the healthy participants only show a 0.1 difference on a -1 to 1 scale, the post-surgery patients can show differences of well over 0.2. The impairments seen at both patient groups is predominantly to the contralateral side. The majority of patients do not show asymmetries that are distinguishable from the healthy participants.

**Fig 11:**
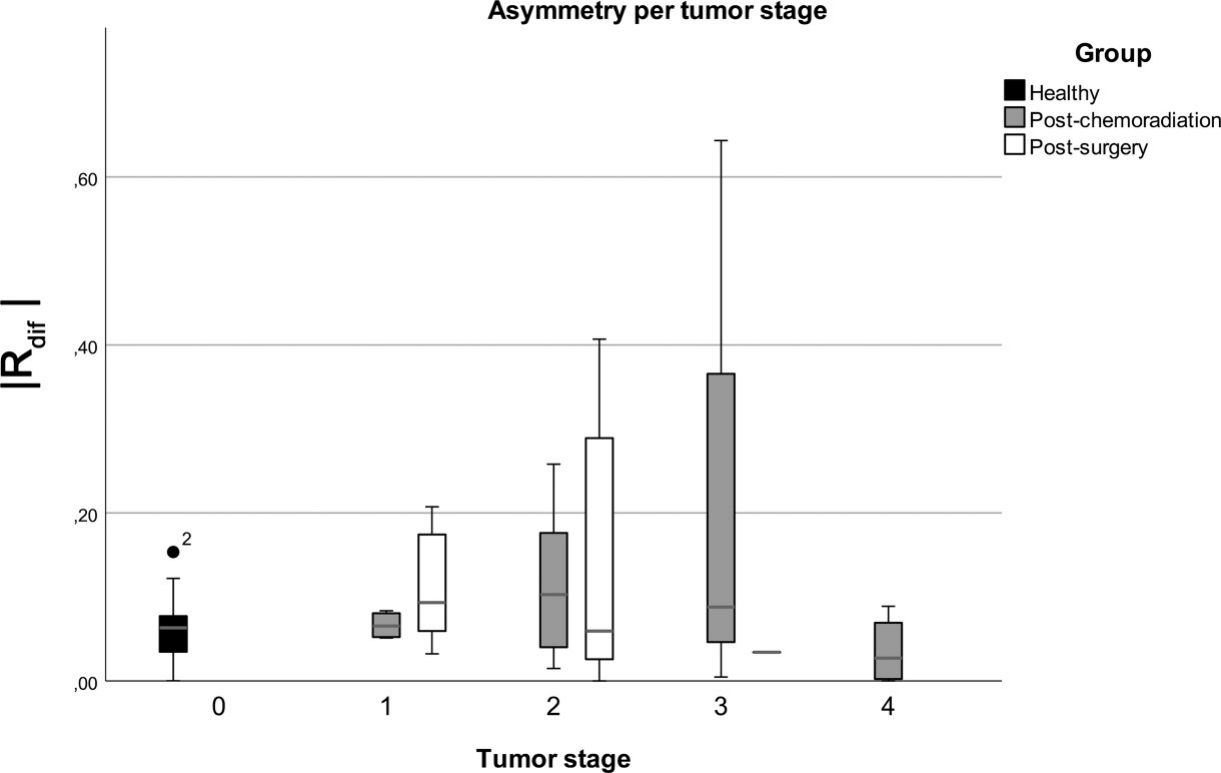
Absolute normalized difference between ipsilateral and contralateral range (|*R*_*dif*|) grouped in T-stage and participant group. The healthy participants group is black, post-chemoradiation is grey and post-surgery is white. The median is shown in dark-grey. There was only one T3 post-surgery patient which is shown as a small stripe. Created using SPSS statistics version 25 (IBM).

To visualize the influence of tumor stage, the absolute differences between the normalized ipsilateral and the contralateral range (|*R*_*dif*_|) are shown in boxplots per T stage in Fig 11. The boxplot shows that the median of the difference does not differ much with increasing T-stage, but it shows an increased variability and an extended upper quartile of the boxplot that is especially predominant within the patient groups. Four patients with T4 tumors treated with chemoradiation showed no asymmetry.

Fig 10 shows that in patients with asymmetrical movement the impairment is predominant on the contralateral side for a small number of patients. Since the range is now measured over an axis using a coordinate system, it is not possible to compare this data directly to our former study wherein only the Euclidean distances were compared. To show that the post-surgery group is comparable to our former study we calculated the Euclidean distances between the two central incisors and the tongue tip for both the ipsilateral and contralateral movement [15]. The results in Table 3 show that the mean Euclidian distance of our patient population is 2 mm smaller in both directions compared to the former study. While showing a significant difference between ipsilateral and contralateral, the Euclidian distance is still highly dependent on the initial pre-treatment ROM, and is therefore not as trustworthy as normalized measures.

**Table 3:** Comparison of the mean Euclidian distance for ipsilateral and contralateral movement between the current study and the former study of van Dijk et al[15].

Euclidian distance	Current study (N = 17)	Former study (N = 10)
ipsilateral	45.6 mm(7.9)	47.5mm (7.0)
Contralateral	40.8 mm(7.1)	42.2mm (5.8)
p-value	0.016	< 0.001

## Discussion

Tongue mobility impairment, for which ROM is an objective measure, has proven to be an important estimate for oral functions like speech[12]. A reliable method is therefore essential for both research and clinical practice. By elaborating on previous work, we created a method to track tongue tip trajectories and put these in perspective by introducing a coordinate system based on facial features[15]. This coordinate system enables localization of the tongue tip at every moment during tracking. By also compensating for head and jaw motion, this method proves to be a robust tool to measure the ROM more accessible and easier to use than other common techniques such as video fluoroscopy, CT, MRI, Ultrasound or EMA.

The range and deflection angle extracted from the 3D tongue tip trajectory showed excellent reliability with ICC’s above the 0.9, which is the same as or former study[15]. The repeatability experiment showed an overall small standard deviation of 2.5 mm to 3.5 mm for every movement, except for the upward movement. We experienced that the up movement was hard to perform and to reproduce for the healthy participants and impossible to perform for about half of the patients.

In addition, other indicators such as volumes, areas and more indicators from the Y and Z axis, which could be derived from the trajectories, were considered. While these could be of use when comparing the ROM of a single participant over time, it showed no additional value when comparing indicators between participants and patients using our current post-treatment dataset. Furthermore, measurement errors will rise exponentially when indicators are derived from multiple measurement points.

The addition of the jaw-movement compensation lowered the standard deviation for the range indicators and increased the standard deviation of the deflection angle by a small amount. While the differences in standard deviation are very small, the benefit of adding jaw-movement compensation is large, because some participants displayed more inherent jaw movements than others. This was mainly visible in compensatory behavior in healthy participants and patients with a very small ROM. A limitation of the jaw compensation was that some participants were able to cover their mandible area during the downward movement for which manual adjustment was needed.

In order to translate the tongue tip trajectory to interpretable results, we introduced a coordinate system. We choose to determine the coordinate system based on the maxillary incisors and the caruncles of the eyes because these points are fixed facial features that will retain the same location after tongue cancer treatment. This makes this system particularly suitable for repeated measurements of a single individual over time. The limitation, however, is that the orientation of the coordinate system will differ between individuals, as the eyes and the maxillary incisors are not aligned at the same angle. At this moment, there is no definitive solution to this problem. However, this will not influence the horizontal moments, which will, therefore, be more suitable to compare between individuals.

While large parts of the method are automated, some key parts of the method still depend on human interaction. During the acquisition, variation could be induced by misplacing the marker, insufficient instructions or non-compliance by the patient. In addition, the observer is responsible that the automatic tracking is performed properly. We found that in some cases the tracked 3D position drifted off over time due to small discrepancies between the tracked location in the three videos. A small error in one or two of the three videos can lead to a misinterpreted 3D location of the landmark, mainly in the transverse plane (or Z-axis). In a future release aimed at performing measurements in a clinical setting, an automated feedback system could inform the observer about significant back-projection errors. The system could also be improved by adding more cameras at different heights.

Tracking a single point in space is very quick, convenient and inexpensive. The measurements performed on patients were finished within minutes without any discomfort. This is an advantage in comparison to EMA which takes time to set up, is expensive, and uncomfortable for patients. However, measuring only one point has its limitations. The shape and position of the rest of the tongue remains unknown, which makes a ROM measurement at a single moment less useful speech and swallowing analysis. The ROM measurements are most useful when measured over a period of time to quantify the improvement or deterioration of tongue motion.

### Comparison with clinical expectations

Tongue movement varies greatly between participants. In our study, the range from left to right (*R*_*total*_) within healthy participants varies from 60 mm to over 100 mm (Fig 7). Because of this variation, it is not possible to distinguish an individual patient from a healthy participant purely on the range or deflection angle; but as a group, they are clearly distinguishable in Figs 7 and 8. We can, therefore, assume that in general, a post-surgical patient not only has a smaller range, which was expected based on previous research but also has a larger deflection angle compared to healthy participants. The post-chemoradiation patients are in between those two groups, which was expected based on the fact that no tissue is removed and that these tumors involve the base of the tongue, that more often results in problems with swallowing rather than problems with lateral movement of the mobile tongue[3,11].

However, using only post-treatment data, a fair comparison was only possible by comparing the contralateral and ipsilateral properties between participants. Based on previous studies we hypothesized that impaired motion to the contralateral side would be predominant[15]. In Fig 10 the largest impairments are seen when moving to the contralateral side, however, this is not always the case. Only four post-surgery and three post-chemoradiation participants showed a serious contralateral impairment. No dominant impairment to a side is visible when looking at the difference between deviation angles (*φ*_*dif*_) in Fig 9. This is in line with studies that show that the side affected by the defect does not matter or not always lead to lateralization problems[6,13]. However, when calculating the Euclidean distance for the left to right movement, a significant contralateral impairment, comparable with our previous study, is visible (Table 3) [15]. We hypothesize that the combination of the contralateral range and angle result in a mean Euclidean contralateral distance that is significantly different from the ipsilateral distance. However, based on the variation seen in the other Figs (Figs 7, 8 and 9) we assume that this is the case for only some post-surgery patients.

It is clear that some parameters are responsible for the large variation between the three groups. The upper quartile in Fig 9 reflects that a substantial amount of patients have an increased asymmetric movement of the tongue with increasing T-stage, which was expected based on literature[33,34]. However, while the variation in asymmetry increases with T-stage and type of treatment, the median does not, which is largely in line with the rest of the results. The same can be found in other literature such as Zuydam et al.[8] where speech scores after surgery are overlapping between T-stages or did not yield significant correlation with speech function[8,10]. Furthermore, it is shown in previous literature that post-operative impairment not only depends on the size and location of the treatment but also on the amount of scar tissue and compensatory tongue motion patterns[12,14]. In the case of a T4, the tumor usually also involves other tissues in the oropharynx where we cannot account for. The inclusion of these parameters would require a larger study population and would be outside the scope of this paper. Furthermore, data about the resection volume and location that were collected retrospectively are rarely precise. Future studies should focus on collection of detailed information on size, tissue-, muscle- and innervation-properties of the tongue. To analyze the effects on ROM in detail, location and size of the treated area of the tongue in patients should be included as well. Also, pre- and post-treatment measures of swallowing and assessment of speech quality and intelligibility can be used to asses if ROM is a valuable tool for the prediction of function loss.

## Conclusion

We elaborated on previous work of van Dijk et al. (2016)[15] to introduced an improved reliable and reproducible method to measure the ROM and to quantify for motion impairments as a fast, secure and accessible alternative for classical imaging techniques and EMA. Using this method, the exact location of the tongue tip can be tracked throughout different tongue maneuvers, while also compensating for head and jaw movement, and thus extending the possibilities of ROM measurements. This way of objectively obtaining the ROM of the tongue tip is essential for methods aimed at predicting treatment outcome, such as biomechanical prediction models, as an addition to the shared decision making in treatment choices. Moreover, it would also greatly improve the objectivity of determining progress during logopedic treatment and rehabilitation in which improvement lingual mobility is the primary focus[16].

With this improved method, we explored the various indicators from which tongue range and deflection angle could be explored and validated using our dataset. From the post-surgery and post-chemoradiation patients, only a small part showed asymmetrical movements, which could not always be explained by T-stage or the side of treatment alone. Future studies should focus on measuring ROM in patients with oral cancer pre- and post-treatment in combination with functional measures and detailed characteristics of the treatment to show if a change in ROM is predictive for functional loss.
